# The gendered impact of Buruli ulcer on the household production of health and social support networks: Why decentralization favors women

**DOI:** 10.1371/journal.pntd.0007317

**Published:** 2019-04-15

**Authors:** Ines Elvire Agbo, Roch Christian Johnson, Ghislain Emmanuel Sopoh, Mark Nichter

**Affiliations:** 1 Faculté des Lettres Arts et Sciences humaines, Université d’Abomey Calavi, Abomey Calavi, Benin; 2 Centre de Dépistage et de Traitement de l’Ulcère de Buruli, Allada, Benin; 3 Centre Interfacultaire de Formation et de Recherche en Environnement et Développement, Université d’Abomey Calavi, Cotonou, Benin; 4 Institut Régional de Santé Publique, Université d’Abomey Calavi, Ouidah, Benin; 5 School of Anthropology, University of Arizona, Arizona, United States of America; Swiss Tropical and Public Health Institute, SWITZERLAND

## Abstract

**Background:**

Buruli ulcer [BU] is a chronic and debilitating neglected tropical skin disease caused by *Mycobacterium ulcerans*. The treatment of moderate to severe BU affects the well-being of entire households and places a strain on both gender relations within households and social relations with kin asked for various types of support. In this paper, we employ the conceptual lenses provided by the Household Production of Health approach to understanding the impact of illness on the household as a unit of analysis, gender studies, and social support related research to better understand BU health care decision making and the psychosocial experience of BU hospitalization.

**Methods:**

An ethnography attentive to circumstance and the nested contexts within which stakeholders respond to BU was conducted employing semi-structured interviews, illness narratives, and case studies. An iterative process of data collection with preliminary analyses and reflection shaped subsequent interviews. Interviews were conducted with 45 women in households having a member afflicted with BU in two communes of Benin with high prevalence rates for BU. The first commune [ZE] has a well-established decentralized BU treatment program and a well-functioning referral network linked to the Allada reference hospital specializing in the care of BU and other chronic ulcers. The second commune [Ouinhi] is one of the last regions of the country to introduce a decentralized BU treatment program. A maximum variation purposeful sample was selected to identify information-rich health care decision cases for in-depth study.

**Principal findings:**

Study results demonstrated that although men are the primary decision makers for healthcare decisions outside the home, women are largely responsible for arranging care for the afflicted in hospital in addition to managing their own households. A woman’s agency and ability to influence the decision-making process is largely based on whatever social support and substitute labor she can mobilize from her own network of kin relations. When support wanes, women are placed in a vulnerable position and often end up destitute. Decentralized BU treatment is preferred because it enables a woman to remain in her own household as a patient or caretaker of an ill family member while engaging in child care and petty revenue earing activities. Remaining in the hospital (a liminal space) as either patient or caretaker also renders a woman vulnerable to rumor and innuendo about sexual liaisons and constitutes a form of social risk. Social risk in some cases eclipses the physical risk of the disease in what we would describe as a hierarchy of risks.

**Conclusion:**

This study illustrates the importance of decentralized treatment programs for NTDs such as BU. Such programs enable patients to remain in their homes while being treated, and do not displace women responsible for the welfare of the entire household. When women are displaced the well-being of the entire household is placed in jeopardy.

## Author summary

In this gender-focused study of the neglected tropical disease Buruli ulcer (BU) in Benin, West Africa, we document how seeking care for BU is influenced by broad-based concerns about the household production of health and the availability of resources women can mobilize from their social support networks. Women and girls shoulder a disproportionate share of the burdens incurred by BU treatment and prefer decentralized treatment from local health stations to free hospital care. Long term and often-indeterminate residence in hospital threatens the integrity of households and results in marital stress, economic vulnerability, school and vocational training dropout, and loss of essential income-generating activities. The case study of BU clearly demonstrates the necessity of recognizing the household, and not just the patient, as a unit of analysis in public health and the need to consider the ripple effect of serious illness beyond the household to one’s social network. We draw attention to the fact that while men are the decision makers about health care in patrilineal Beninese society, a women’s agency in influencing decision making is tied to her accumulation of social capital, capital that is taxed by long term medical treatment weakening her safety net in the future.

## Introduction

Much has been written about the health care seeking process in low and middle income countries (LMICs) and the predisposing, enabling, and service-related factors that contribute to health care decision making for different types of health conditions and diseases[1, 2]. Studies have also addressed how cultural perceptions and past interactions with practitioners affect present and future health care actions in a pluralistic health care arena. What has been underrated is how households cope with the direct, indirect, and opportunity costs of health care and the impact of illness on not just the afflicted, but other household members and the members of one’s broader social network[3–6]. A more complete understanding of health care decision making demands greater attention to the household production of health, gender relations, the mobilization of therapy management groups, and the ripple effect of illness on social support networks.

Adopting a household production of health (HHPH) approach to decision making[7, 8] situates health care within the full range of activities undertaken to achieve well-being for the household as a unit of analysis. Well-being extends beyond physical health to considerations of social relations, moral identity, and psychological health. Notably, this approach considers a household’s selective investment of time and limited resources, the trade-offs it makes when addressing pressing needs and real-world contingencies, and the opportunity costs of different courses of action.

Anthropologists have drawn an important distinction between the household as a structural and a functional unit[9]. An HHPH approach favors a functional, task oriented definition of the household that privileges the processual study of how health is produced, promoted, maintained, and protected by household members defined less by cohabitation (structural criteria) and more by routine participation (functional criteria) in health/well-being related activities. Households can include kin [and fictive kin] who are working or living elsewhere, but contribute to the household in some way, especially at times of urgent need, and who derive part of their identity by an affiliation to the household.

Social scientists studying the household as a unit of analysis are well aware that relations within households are both competitive and cooperative at different times, that the social status of members is not equal, that status changes over time according to varying criteria (e.g., age, work, financial contribution, marital status), and that intrahousehold negotiation between men and women over the use of resources takes place in subtle ways [10, 11]. Times of sickness in households with scarce resources are often occasions when tensions run high, especially when health care decisions implicitly or explicitly (dis) favor particular household members or courses of action. Decisions often take place in the context of ambiguity, do not reflect consensus, and are contingent.

Gender has been recognized as an important factor in studies of the HHPH, health care decision-making, and the allocation of scarce resources in times of sickness [12–14]. However, good case studies that illustrate different ways in which gender roles and relations within a household are affected by illness in LMICs, especially longstanding and chronic illness, are rare. Needed is research that examines differing demands treatment places on men and women during different points in an illness treatment trajectory. Given that women typically attend to the ill, special attention needs to be focused on economic, social, and affective challenges to women tasked with being caretakers for both children and the ill, and the ramifications of health care decisions.

A third dimension of health care decision-making addressed in the literature is social support. Of particular importance is the mobilization of therapy management groups (TMGs) from within one’s larger support networks. TMGs are the constellation of individuals who take charge of various aspects of therapy management with or on behalf of the afflicted[15,16]. They are composed of all members of one’s social support network having an impact on any aspect of health care decision-making, care seeking, and support. Members may include kin, friends, community health workers, health staff, and traditional healers. In short, the TMG is composed of everyone who weighs in or contributes to health care in some way. TMG address many “works of illness” from decision making and economic assistance to substitute labor and psychosocial support [17].

To date, few studies have addressed gender and temporal dimensions of TMG mobilization. Men and women have different social support networks and resources to draw upon. We know far too little about who each turns to, for what, and with what expectations. We also know little about how the composition of TMGs change over time and the degree to which levels of support are responsive to competing demands on member’s time, resources, and other social obligations. Missing in the therapy management literature is adequate consideration of the temporal dimension of TMGs mobilized to respond to longstanding and chronic disease. Also missing are studies that address reasons for TMG failure and patient abandonment.

In this paper, we employ the conceptual lenses provided by HHPH, gender studies, and social support related research to better understand health care decision making for Buruli ulcer (BU), a neglected tropical skin disease endemic in West Africa.

Buruli ulcer is a chronic, debilitating disease caused by *Mycobacterium ulcerans*[18]. It usually manifests through non-ulcerated lesions such as nodules, plaques, or edema that may evolve into massive skin ulcerations, joint and bone deterioration if left untreated[19]. Most cases of BU are found in West Africa and Benin is one of the endemic countries[20].

Fifty percent of those afflicted with BU are adults and 50% children. Most of those afflicted experience lesions on their limbs, although lesions may appear any place on the body[21]. The disease is non-contagious, the route of BU transmission unknown, and its incubation period poorly understood[22, 23]. The poorly understood transmission of the disease and the fact that scattered households, not clusters of households, are typically affected has reinforced local speculation about BU related wounds being possible signs of supernatural contact or witchcraft.

Up until 13 years ago, the management of BU required surgical removal of all sites of infection. In 2004, antibiotic treatment was found effective at early stages of the disease (category I: lesions < 5cm in diameter; and category II: lesions between 5 and 15cm in diameter)[19]. At present, the management of BU has three main components. Antibiotic treatment is based on daily oral rifampicin (10 mg/kg) and streptomycin (15 mg/Kg) injection for 56 days, which allows lesions whose diameter is less than 10 cm to heal without surgery[24, 25]. Effective outpatient antibiotic treatment at early stages reduces wound dressings and avoids skin grafts, which are needed for large ulcerations. More advanced cases often require long-term hospital treatment of indeterminate duration and physical therapy to prevent disability, amputation, and functional limitations after care[26]. Treatment for BU is provided free in most West African countries either at hospitals (centralized in-patient care) or at local health stations (decentralized outpatient treatment care).

Studies of BU in West Africa have found that biomedical treatment for BU is often delayed for reasons linked to perceptions of causality, fear of surgery and amputation, and the logistics and costs of seeking “free care,”[27]. With respect to cost, several studies [4, 28–30], have drawn attention to indirect and opportunity costs of “free medical care” to households. BU provides an excellent opportunity to address limitations in the health care seeking literature highlighted above. More specifically, it provides an opportunity to more closely examine both how households and social networks are affected by hospital based medical treatment of indefinite duration, and risks to female patients and patient caretakers.

## Methods

### Study site

The study took place in Benin West Africa. Benin is bordered by Togo to the west, Nigeria to the east, and Burkina Faso and Niger to the north. The country is highly dependent on subsistence farming, regional trade, cotton as a cash crop, and remittances from seasonal migrant work largely to Nigeria. Over twenty different sociocultural groups inhabit Benin, the vast majority of which are patrilineal, meaning that children are part of their father’s lineage. Women typically maintain close ties with their own female kin. While both men and women contribute to household economics, women are largely responsible for providing resources for routine household needs. Women generally do so through the cultivation and sale of agricultural products as well as petty trade. Microfinance schemes for women are available in some, but not all regions of Benin.

Benin is one of the most endemic countries for BU in West Africa [20]. Benin is divided into four regions and twelve *departments* subdivided into 77 communes. The National Control Program for BU in Benin supports four reference centers (CDTUB) located in Allada (Atlantic region), Lalo (Couffo region), Pobè (Ouémé region), and Zagnanando (Zou region). Each referral center supports a number of peripheral health centers that provide decentralized case management [31]. The mission of peripheral centers, which are state run health stations, is to provide accessible care for simple cases of BU (category 1 and 2). Reference centers, like the Catholic mission hospital of Zagnanado, are in charge severe cases. Field sites chosen for this study were located in two regions with high BU prevalence rates [31]: the Atlantic region (Zè commune) and Zou region (Ouinhi commune). Decentralized management of BU patients is well established in the Atlantic, Ouémé and Couffo regions of Benin. In these regions, most cases of mild to moderate BU (Category I and II) are treated at health stations staffed by nurses. More serious (category II and III) cases are referred to reference hospitals. Decentralized treatment of BU has only recently been introduced in the Zou region[31]. Up until 2016 when a pilot decentralization project was initiated in Ouinhi commune, BU patients in the region were served almost entirely by a Catholic mission hospital renowned for surgery-based treatment for all cases (category I, II, III) of BU [31]. Zou region has only begun the process of adopting decentralized BU treatment. In Ouinhi commune, only one of four health stations are presently treating BU cases. As noted in an earlier publication [31] this health station become very popular and is receiving patients who had previously refused to be treated in hospital.

### Study design

A circumstantial ethnography[32] was conducted employing semi-structured interviews, illness narratives, and case studies. A circumstantial ethnography focuses on how nested sets of actors influenced by differing life circumstances respond to a focal phenomenon, in this case the treatment of BU. The ethnography was attentive to the experiences of patients and caretakers as well as responses of therapy management group members responding to requests for support. The study design allows for an iterative process of data collection with preliminary analyses and reflection shaping subsequent interviews. Case studies were collected using a “life history” approach, which focuses on the interviewee, and their storytelling to understand how perspectives and discourses are constructed [33]. In the present study, the focus was on the experiences of women deliberating and reflecting on BU treatment decisions, institutional care, household survival issues, and social support relationships. A narrative approach was chosen in which the focus is on people’s evaluations of their own life experiences [34].

A maximum variation purposeful sample[35] was selected to identify information-rich experiences for in-depth study. Interviews were conducted with 45 women who were either afflicted with BU themselves, caretakers for a family member with the disease, or the decision maker for whom in a household should accompany a patient to the hospital. One man who had uncharacteristically taken on the task of managing his son’s BU treatment was also interviewed. The sample included women whose husbands resided for most of the year in their homes and women whose husbands were migrant workers, married and widowed women, and women faced with managing moderately severe and more advanced cases of BU in the hospital (centralized treatment) and by daily visits to a health station (decentralized treatment). Informants were identified with the help of community health volunteers and clinic staff in community, clinic, and hospital settings in both Ze and Ouinhi communes. Hospital patients included both residents from the region in which the hospital was located, and patients and caretakers traveling to the hospital from outside the region. Once community health workers and health care providers identified people afflicted with BU, they were contacted and asked to participate in qualitative interviews about their illness experience.

The principles of thematic narrative analysis were followed [36]. After re-reading interview transcripts, the findings of interviews and narratives were discussed by team members, and coded for both focal and emergent themes. Focal themes included predisposing, enabling, and service related factors influencing BU treatment decisions, gender relations, social support, choice of patient-caretakers, and patient abandonment. Emergent themes include rumor and social risk, quality of childcare, and impact of treatment on children’s schooling. After an extensive consideration of the data obtained along, short vignettes and interview extracts in line with the study’s objectives were chosen as exemplars for use in this publication. Vignettes chosen illustrate the backstage of treatment decision-making and care management along with the complexities and contradictions revealed by a study of real-life circumstances[37]. Themes introduced in the results section provide answers to core research questions posed as a heuristic [38].

### Ethical approval

Ethical approval was obtained from Benin’s National Ethical Committee of Health Research before the start of the research (IRB00006860 N° 148 /MS/DC/SGM/DFRS/CNPERS/SA). Informed consent procedures already in place at Allada hospital were strictly adhered to over the course of the project. All patients and staff interviewed were assured that interviews would be kept confidential. The use of oral consent was approved by the ethical review board because many study participants were illiterate. When a participant was under 18 years of age, both the child/adolescent and his/her caretaker were informed about the nature and aim of study before being asked to give oral consent.

## Results

The results of our ethnographic study are presented as responses to seven key questions posed as a heuristic: 1) Who makes decisions about when and where to receive BU treatment; 2) What core HHPH concerns influence health care and patient caretaker decisions; 3) How do women influence BU related decision making; 4) What is the ripple effect of BU beyond the household; 5)What leads to abandonment during hospitalization; 6) Why are widows and their children a group at risk; and 7) Why do households, and particularly women, prefer decentralized treatment for BU. Data presented in response to the first six questions enable a nuanced response to the seventh question and provide valuable insights into community response to “free” BU treatment. Case vignettes are provided as a means to give a human face to core issues being highlighted and brief reference to Pan-African themes are noted to situate research results in a larger context.

### Who makes decisions about when and where to receive BU treatment?

In Sub-Saharan Africa, gender roles and social norms of seniority and power strongly influence how health care decisions are made [39–43]. In our research sites, most ethnic groups are patrilineal. Health care decisions that entail treatment outside of the home are made by husbands or senior members of their kin network. This is true even if a husband is employed as a migrant worker and absent from home much of the year. In our sample, 21 women acted as heads of their household during all or much of the year. Only two women reported making a BU related health care decision on their own. Women followed the health care advice of a husband or his kin regardless of whether they offered any financial support for BU treatment. Our informants noted that if a woman did not seek approval from her husband or senior members of his family, she left herself open to social censure. In some cases, however, a husband and his kin abandoned a sick child, an issue we will address shortly.

### What core HHPH concerns influence health care and patient caretaking decisions?

It has been widely reported in studies of health care seeking in West Africa that enabling factors are as important as predisposing factors (such as perceived cause) in determining when and what kind of health care is sought [6, 27]. Our study corroborated this finding. The enabling factors most commonly referenced in interviews about hospital-based BU care were the indirect costs of “free treatment” such as transportation costs, food and incidental costs (soap, mobile phone credit, etc.), and the opportunity cost of lost labor. Decision makers (husbands, elder kin) took stock of available sources of substitute labor within the household as well as a wife’s social capital, her ability to mobilize support from her own kinship network.

A mother’s absence from home on a daily basis to obtain outpatient treatment for BU or her need to remain at a hospital as either a patient or caretaker was deemed feasible only when essential household duties were taken on by somebody else. Women’s labor demands varied by season and household composition and encompassed agricultural labor, cooking, securing water and firewood, and childcare. Daughters were generally turned to first to take on a mother’s responsibilities in the household or to serve as a caretaker for a hospitalized family member. When a mother did not feel it was safe to leave small children at home to be cared for by an older child, or her labor in the fields was required for household survival, a daughter was commonly sent to care for a sibling in the hospital. This often interfered with her own schooling or apprenticeship activities. If, on the other hand, a mother was the patient, a daughter was sometimes asked to take charge of household duties in her absence. The following cases illustrate the complexity of patient caretaker deliberations as an important factor in health care decisions, and the role children play as patient caretakers given household production of health concerns.

**Madeleine** (daughter, patient), aged 11, was admitted to Allada hospital for treatment of BU after initially receiving decentralized treatment at a health station near her village. Her mother suffers from poor health, making it difficult for her to manage the household and tend to the fields. As a result, her husband took on a co-wife, who has three children of her own. Madeleine’s mother was afraid to accompany Madeleine to hospital and leave her other four children at home under her co-wife’s charge as she suspected they might be mistreated. Madeleine’s mother received assistance from her own mother and two sisters when she took Madeleine for decentralized care at a local health station a few kilometers away. However, when the child’s wounds did not heal, they were reluctant to offer long-term support for Madeleine if she was hospitalized. Madeleine’s father decided that the best option was to send Madeleine to the hospital along with her 8-year-old sister, Reine. Reine was taken out of school to care for Madeleine. Madeleine’s two older brothers were not asked to be a caretaker as this was seen as women’s work. Both Madeleine and Reine wished to continue their education, but their mother recognized that this was unlikely if long term BU treatment was required. In effect, Reine’s future was sacrificed to attend to her sister.

**Clemency (**mother of three, patient) needed to be hospitalized for an advanced case of BU, but she had no adult family member able to provide support. Her own mother was deceased and her two sisters were working in Nigeria. It was decided that Clemency’s teenage daughter would remain at home to tend to the household and that her younger, five-year old daughter would serve as her caretaker in the hospital. Her husband agreed to supply necessary resources during treatment. Clemency entered the hospital with her five-year old daughter and her 18-month-old son. Clemency required several surgeries and was confined to bed and a wheel chair. Her five-year-old daughter performed all tasks necessary for their survival in the hospital including going to the market, cooking, washing clothes, taking care of her baby brother, and making sure her mother took her medicine on time. Clemency’s daughter was helped by other caretakers and nurses in the hospital who spoke of her with great admiration. One often saw Clemency’s daughter going about her business with her younger brother on her back. Her mother described her daughter as a gift from God. However, she worried about her future, especially her schooling. She noted: “I do not know when I will finish with this treatment, no one tells me. If I can finish in a few months then my daughter will be able to go to school and can catch up. But, if I have to remain in the hospital longer, what will happen to her? While she is very intelligent, it will be hard for her to succeed in school.” As in the case of Reine, the future of a young patient caretaker was placed in jeopardy as an opportunity cost of treating a sibling afflicted with BU in hospital.

Two other household production of health issues emerged in BU illness narratives that are rarely discussed in the health care seeking literature. The first is a mother’s concern about the quality of childcare in her absence. This psychosocial concern sometimes eclipsed concerns about a child’s physical condition. The following case illustrates the importance of the quality of childcare in health care decision-making. The case involves a decision to decline free hospital treatment for a child afflicted with BU.

**Prisca (mother, caretaker)** is the sole resource-provider for her household. Her husband works, but most of the money he earns is spent on sodabi palm wine. One of Prisca’s children, an 11-year-old daughter, suffers from advanced (category II) BU, which requires hospitalization and possibly surgery. At first, Prisca administered home treatment to her daughter. When her lesions grew in size, Prisca asked permission from her husband to seek outpatient treatment for her daughter from the district health center 4 KM away. This proved challenging as Prisca still had to find the means to support the household on a daily basis through petty trade. After two months of treatment at the health center, her daughter’s condition was still serious and health staff referred her to Allada hospital, where she could receive free treatment.

At first Prisca refused to take her daughter to the Allada hospital even though she was concerned about the size of her lesions. Health staff and a doctor from Allada visited Prisca and attempted to change her mind, but she did not agree, stating she had no one to look after her other young children. She did not feel secure leaving her children in the hands of her husband. She stated, “Seeking care at the district health center is possible because it does not prevent me from going about my business and ensuring the well-being of everyone. Leaving the house for who knows how long, that is simply not possible.”

A few days later, a social worker from the hospital returned to Prisca’s house and offered to look after her daughter while in the hospital if no family member could accompany her. **Prisca** spoke to her husband, who agreed to allow their daughter to go to the hospital as long as significant cost was not involved. After two days of treatment at the hospital, however, Prisca returned and took her daughter home. When interviewed as to why she did so, the mother stated that her heart would not allow her to leave her daughter in the hands of an unknown woman. She went on to note: “I prefer that my daughter continue with the bandaging at the district health center even if this is not the best treatment. Some infirmity may result, but it is better than the total destruction of all members of my house.” She then when on to state: “When it comes to sickness only a mother can comfort and care for a child properly. In the hands of someone my daughter does not know, she is likely to suffer. How can I have a quiet heart at home worrying about her?”

A second notable concern that we identified as having a big effect on health care seeking and patient caretaker decision making was social risk (risk to reputation and to present and future social relationships) [44, 45]. When a woman leaves the confines of her village either to visit a health post some distance away or to reside in a hospital, she risks becoming the subject of rumors about sexual indiscretion. Such rumors question a woman’s moral identity and a husband’s masculinity and cause strife between husbands and wives. We found this to be a common reason a mother took a child with her when visiting a health post or when residing in a hospital. However, we found that even when a woman brought young children with her to hospital, she was still subject to rumor. Fear of rumor was a constant worry for some women, adding to the stress of social isolation and trying to survive with minimal resources. The following case illustrates how an apparently stable marriage was destroyed by rumor and innuendo:

**Ruth** (mother, caretaker), was given permission by her husband to care for their five-year-old daughter while she was being treated for BU in hospital. Ruth also brought her infant son to the hospital, as she was still breastfeeding. Ruth received regular visits from her husband, who was very attentive to her needs and those of their children. However, during one visit to the hospital he became quite agitated. Late in the evening, he awoke to see someone enter the ward, approach the bed of a young female patient, hold her hand and kiss her before departing. This event shocked her husband and he began to suspect his own wife’s fidelity. He began to see the hospital as a site of moral dissolution where patients and caretakers engaged in extramarital behavior. Without evidence of any wrongdoing on the part of his wife, he took the extraordinary measure of abandoning his wife and small children. When interviewed, he remained resolute, exclaiming, “These doctors, they may bring healing, but they destroy homes!”

Fear of rumor influenced who was chosen to be a patient caretaker in hospital. Daughters who had not yet reached puberty were preferred. The hospital was seen as a liminal space and time in the hospital to be quite boring. Several informants noted that “people” suspect that any young woman with limited resources will engage in sexual relations if outside the watchful eye of community members. We recorded cases where a daughter as young as 15 was sent to the hospital as a caretaker only to be returned home when rumors about sexual relations emerged. The following is an example:

**Florent** (son, patient) aged 18, was admitted to the Allada center for BU treatment. **Florent** is the third of seven children. His father lives and works as a brick maker in Nigeria with one of Florent’s brothers. Florent’s older sister, an apprentice seamstress, was asked to leave her apprenticeship to be his caretaker at hospital. Florent’s father suggested this course of action given that there were young children at home that needed their mother’s care.

During Florent’s hospitalization, his mother heard a rumor that Florent’s sister charged with his care was becoming romantically involved with men at the hospital. Fearing that her daughter’s reputation might be spoiled or that she might become pregnant, Florent’s mother sent her daughter back to her apprenticeship and replaced her as Florent’s care provider. This necessitated bringing four of her children with her: her two-year-old daughter and three children who had been attending elementary school.

Taking care of young children in the hospital wards is not easy for Florent’s mother. Her husband supports her, but sends money irregularly and what is sent is not enough to meet their needs. Because she can no longer work in the fields or engage in petty commerce in her village, Florent’s mother tries to make money any way she can while in the hospital by washing clothes, cleaning, and running errands for staff and other patients.

### How do women influence decision-making?

As has been noted elsewhere in sub-Saharan Africa[46], although women do not have the same kind of authority as men, it would be misleading to present them as having no impact on health care decision making. Most women we interviewed asserted that although men have the final say in decisions about health care, women’s input and counsel influence decisions. Women typically accepted their husband’s initial health care decision, even if they did not agree with it. However, they often encouraged husbands to reconsider decisions based on shifts in disease trajectory as well as the availability of different types of material and social support. And in a few cases, they took matters into their own hands when they felt they were being abandoned by a husband and his kin.

Also, as in other parts of Africa [6, 47], we found that women’s agency in health care decision-making was largely based on two things: the resources she has at hand, and her ability to mobilize resources from kin in the form of material goods, labor, and childcare. The best way a woman could influence BU-related health care decisions was by working out how a treatment option she favored could take place with only minimal disturbance to essential household production activities. Having a daughter, as noted in the cases of Madeleine and Clémency, was an asset. If one’s own daughters were old enough to serve as the caretaker of a sick family member, or to remain home and assume household responsibilities, a mother had some flexibility. However, when a woman did not have a daughter to assist her, she was compelled to approach kin and ask them for support and to play a more active role in therapy management.

Based on our data, most of a woman’s requests for assistance were to her own mother and sisters, followed by friends and neighbors. Asking members of her husband’s family for assistance was only a last resort. The following case illustrates kin coming to the aid of a sick relative wanting to be treated in hospital for BU and in need of a caretaker. In this instance, a niece was removed from vocational training to care for her aunt and as a result experienced biographical disruption, an interruption and destabilization of the life trajectory of the caretaker [48].

**Gisèle** (caretaker), aged 23, has been the caretaker for her aunt in Allada hospital for the last 19 months. Her aunt’s wounds form BU are quite serious and her treatment is likely to go on for some time. Prior to coming to the hospital, Gisèle was an apprentice seamstress attending a vocational training course. She planned to open up her own small tailoring shop soon after graduation. Gisèle was asked by her mother to take leave from her tailoring course to care for her aunt while in hospital. Her aunt had assisted their family in the past and she had no daughters of her own to ask for help.

When interviewed, the first thing **Gisèle** said was that she had never imagined how much her life would change when assuming a caretaker role in the hospital. She did not resent taking care of her aunt, but was sad about her fate stating that her “heart was in a vice.” She noted “I agreed to stay with my aunt because she is like a mother to me. She has always helped my family. But, by being here I have lost many things. I have no income-generating activities here. I have lost both financially and professionally. I was at the end of my apprenticeship and I was working to raise money necessary to obtain my diploma. My classmates have already graduated and they are now employed, but I am here. I worry about losing my tailoring skills, and I worry how I will raise money for my graduation. I try to find small jobs in the hospital, but whatever money I make is spent on food. My boyfriend has also become distant. He came here once and saw a male nurse teasing me, and he now suspects that I found a ‘doctor.’ I call him and he does not pick up the phone.”

### What is the ripple effect of BU beyond the household?

As noted in the case of Gisèle, BU hospitalization does not just affect members of one’s immediate household; it also affects one’s broader social support network. In short, asking for and receiving support from kin in times of illness creates a ripple effect. For women living on the margin and having multiple work responsibilities of their own, assisting kin (and fictive kin) out of friendship or obligation is an effective means of reaffirming and strengthening reciprocal exchange relations. Volunteering to take children afflicted with BU to health stations for outpatient care, watching children when a mother is away from home, and lending money or supplying food were all found to be means of solidifying social bonds between women. This form of “bonding social capital” [49–51] provides women with a safety net associated with norms of social reciprocity and cooperation for mutual benefit.

On the other hand, we found that when requests for time or resources exceeded the capacity of kin to provide, social bonds were weakened. The same was true when a mother felt the amount of resources or care provided to her children by kin was inadequate. Requests for long term support often caused conflict within the households of kin. In some cases, there just were not enough material resources to share, and in other cases the opportunity costs of attending to somebody else’s children reduced the time a woman had available to generate revenue needed to support her own household. In short, social capital was a contingent and conditional resource dependent on the presence of resources [52].

Some women interviewed belonged to microfinance schemes and they had to repay loans in order to maintain the integrity of the group. The ripple effect of BU affected the entire group when members were unable to live up to their financial obligations due to the indirect and opportunity costs of BU treatment. The following case illustrates how BU affected one woman’s livelihood and microfinance group membership. Her predicament affected not just her present, but her chances of recuperating economically in the future.

**Juliette** [mother, caretaker] is a food vendor and a palm oil processor who is a member of a local micro-finance group. She contributes to the group monthly to pay off loans she has taken for her business. When her daughter, aged eight, was diagnosed with BU, Juliette took her to the hospital for treatment and resided with her for the next year. Remaining in the hospital disrupted her ability to pay back loans and this affected the entire microfinance group. Even though group members understood that she was caring for a sick child, they pressured her to find money. Her inability to repay loans compromised both the financial standing of the group and her future ability to borrow money. She noted:

“I have so much worry and stress now. What should I do to pay off my debts? The whole village knows that I owe money. I tried to arrange my business affairs from here. I entrusted my aunt with the sale of my goods in order to allow me to pay my debt each month. However, she mismanaged my business. What can I do now? … (she cries). I feel my reputation is now destroyed and I am resented. Women in the microfinance group will not welcome me back into the group. Without a loan, how can I reestablish my business?”

### What leads to abandonment during hospitalization?

As noted by Ribera et al. in Cameroon [53], abandonment is an extreme household coping strategy initiated during catastrophic or protracted illness to avoid plunging a household into a “spiral of impoverishment.” Abandonment was a major concern voiced by our informants. Those residing in hospital as well as those contemplating going to hospital worried they might be abandoned if they remained in hospital beyond the length of time their household could provide for their basic needs. Wives under treatment worried that a husband might find it necessary to take a co-wife to maintain the house in her absence, and husbands afflicted with BU worried that wives might find other men to take care of them in their absence. We documented cases of both scenarios. Hospital administrators in Allada were especially concerned with child abandonment and noted cases where caretakers suddenly just disappeared. They pointed to several abandoned children now residing on the hospital grounds post treatment because they had no place to go. The presence of these children at the hospital was a constant reminder to others of what can happen when household resources are stretched too thin.

When interviewed, women who were abandoned displayed considerable psychological distress related to failed expectations of support in keeping with cultural values based on reciprocity. A common narrative emphasized how much a woman had sacrificed in the past to support other family members in times of need. The following are two examples:

**Honon** (mother, caretaker) is a widow with six children, the youngest of whom are twins. After the death of her husband, his family encouraged her to remarry one of his younger brothers, a proposal that Honon refused for undisclosed reasons. As a result, her husband’s family abandoned her and offered no support for her children. Honon was forced to return to her own family. She and three of her children went to live with her paternal uncle. The other three children were entrusted to other family members in a foster care arrangement (*vidomègon*) common in West Africa wherein children receive care in return for labor. One of her young twins developed BU. Honon’s mother, sisters, and uncle encouraged her to try various types of home remedies for the child. When the child’s wounds became more serious and required hospital treatment, her family members were unwilling to offer support either for Honon to care for the child while in hospital or to care for her other two children in her absence. Her kin felt that the burden of either action would place the household in jeopardy.

Honon was pressured to return the ill child to her deceased husband’s household. This suggestion was quite unsettling to Honon for two reasons. First, her deceased husband’s family had taken no responsibility for his children up to this point in time. She felt that if the child was received, they would be neglected. Second, she strongly suspected that someone in her deceased husband’s family had sent bad luck to the child resulting in wounds that would not heal.

**Honon** stated that she felt abandoned by both her own family and the household of her husband. Against the advice of her own family, she opted to go to Allada hospital and care for her sick daughter. She brought two of her other children along with her as there was no one willing to care for them at home. **Honon** received basic food rations from the hospital and otherwise survived by taking on small jobs when she could find them. She was very bitter about her abandonment. In her own words “Before leaving for Allada my mother promised to come visit me during our stay. It has been 18 months and neither she nor any of my sisters or brothers have visited or contacted me. When my sister was sick and hospitalized, I was the one who had been at her bedside. I was there for so many others in my family in their time of need. For me, no one is offering assistance or showing love. It is as if I am without parents. If it was not for the generosity of the hospital to whom I owe everything, I would be destitute.”

**Conforte** (female adult patient), aged 38, is Togolese and traveled to nearby Benin in search of treatment for an advance case of BU. Her older sister resides in Benin and informed her about Allada hospital. Conforte traveled to Allada and her sister provided her support during the first months of her treatment. However, as the months passed her sister’s resources dwindled and she began to tire of her sister’s illness. Then one day, Conforte noted with bitterness, her big sister stopped coming to visit and would not return her calls. Conforte never heard from her sister again and survived on charity offered to her by her church and hospital staff.

Conforte noted, “When I came here to seek treatment, I gave my big sister all my savings and belongings to hold for me while in the hospital. However, she abandoned me in the most difficult moments of my life. She is the one who asked me to come to Benin. I helped her so much when she faced illness in the past, she felt obliged to support me. It is true that she really helped me during the first months of my hospitalization, but over time, she regretted encouraging me to come here for treatment. She abandoned me. It is nasty, no? When our parents died, I was the one who helped all members of my family until I became ill. Now, where is their support for me in return? Today, strangers help me. In the past, I have helped others outside my family as a good Christian. Perhaps it was the help I gave to others, that led others to help me now.”

### Why are widows and their children a group at risk?

Widows are in a structurally vulnerable position in Beninese society whether or not they agree to a levirate marriage to a brother of a deceased husband who has other wives. In many cases, when a woman with young children is widowed or divorced, she raises her children in the house of her own kin until they are old enough to be sent to the household of their deceased husband. However, as we noted in the case of Honon, if a child becomes ill and is a burden to the household, a widow may be pressured to send the sick child to her deceased husband’s household to bear the costs and responsibility of treatment. If her deceased husband’s family does not offer support, and she opts to bring a sick child to the hospital she may be encouraged to place her other children in foster care as she will no longer be able to provide for them. The following case illustrates the predicament in which many widows find themselves.

**Aline** (mother, caretaker) is a former BU patient herself. When her husband died seven years ago due to an accident, she found herself having to care for their six children on her own. One of her daughters developed BU. When it became clear that she would have to be hospitalized, her deceased husband’s family remained silent about what should be done, and did not offer financial support for treatment. Aline’s mother’s household is very poor and was unable to offer her support. In order to admit her daughter to the hospital, Aline was compelled to place four of her children in foster care in the households of distant kin. This was an act of desperation. **Aline** was upset by the decision, but felt she had no other option.

Aline brought her ill daughter and a young son to Allada hospital. Because of their dire financial situation, the hospital offered Aline’s daughter basic daily food rations. To otherwise survive, Aline’**s** mother (like Honon) is constantly looking for work to support herself. Every day, she sells dumplings to schoolchildren at a nearby school, and gets a small payment in return from the dumpling-makers. The hospital is not happy having caretakers like Aline leave the hospital grounds to engage in petty business, but this is the only way she is able to survive.

### Why do households, especially women, prefer decentralized care?

Our research revealed a strong preference for decentralized BU treatment. Six reasons were identified from interviews that asked women about the advantages of decentralized care. First and foremost, decentralized care does not disrupt a household’s daily routine by removing a mother from her household. Decentralized treatment, for all but very severe cases of BU, still enables a woman to do chores and watch her children as well as engage in entrepreneurial activities essential for household survival. Second, decentralized treatment avoids the many indirect and opportunity costs associated with hospitalization. Third, it allows children to stay in school while being treated, and it reduces the need to remove children from school and apprenticeships to serve as patient caretakers. Fourth, a mother does not have to worry about the quality of sibling care and foster care in her absence. Fifth, she also does not have to worry about pernicious rumors undermining her own or a daughter’s reputation. Sixth, remaining at home during treatment is less stressful. A woman worries less about abandonment.

Fathers who take responsibility for the treatment of a child with BU also favored decentralized care. Although not the focus of our research, we encountered one father who, having refused to send his son to the hospital for BU treatment, agreed to take him for decentralized care. The case illustrates both why he favored decentralized care and why he accepted responsibility for taking his child to a health station. He acted in accord with kinship norms and obligations, and cared for a child from a co-wife he was not currently living with instead of asking a wife home maintaining his home to attend to the health needs of a child by a different marriage.

**Djalil** (father caretaker—outpatient) lives with his first wife and two children in a village in Ouinhi commune. His first wife was infertile and his two sons are children by a second wife, a Nigerian shopkeeper whom he cohabits with while working in Nigeria for some months each year. When his son aged 8 was diagnosed with BU by heath staff attached to the mission hospital he refused to send the boy to the hospital to be treated. He was afraid the boy would have to undergo surgery and remain in an unfamiliar environment for a long time. He was not comfortable asking his first wife to remain with the boy in the hospital. She was not his mother and was involved in petty trade activities that both helped support his household and allowed her to offer some level of support to her mother. It was not possible for him to remain at the hospital, as he too had to work to sustain his household. Djalil also did not want the boy to lose a year or more of school. The boy continued to go to school because his wounds were not painful and his ulcer was not noticeable if hidden under clothing. Djalil initially planned to send the boy to his grandmother’s house in a nearby village during school holidays to be treated by a traditional healer.

As a result of a mass BU outreach education program, Djalil learned about the availability of decentralized treatment at a health station a few kilometers from his house. He consulted the nurse at the health station, who assured him that he could treat his son’s wounds with medicine and bandaging if he adhered to a treatment that required daily visits to the health station for some months. Djalil agreed and engaged himself in agricultural activities at home instead of returning to Nigeria to work. He strictly adhered to the decentralized care offered by the nurse for eight months until his son had fully recovered. In his words, “I was greatly relieved to receive treatment here from the major (nurse) as this type of treatment is not available in the place of his mother in Nigeria. I brought my son to the health station every day for five months. Then, for two months, we visited the station every three days. Finally, in the eight month, I brought him every four days. My son remained at home, surrounded by family, and was able to complete his school year as well.”

## Discussion

Beyond research on BU, this study contributes to the fields of health service research, household and gender studies, and the study of conditional kinship obligations in contexts of poverty where “the capacity to care and the decisions about who undertakes care work are shaped by other considerations: resources and assets that facilitate the work and costs of care; expectations and commitments….; alternative obligations and responsibilities to other house holders; and affective ties to others that do not always follow prescribed kinship ideals” [54]. Geographical accessibility is an important enabling factor influencing healthcare seeking behavior, but as we note in this paper, cultural accessibility and social acceptability also contribute in significant ways. Understanding these factors helps us better understand why decentralized care for BU is preferred, and why centralized care, even when offered for free, is rejected by some households.

Ten important lessons from this study may be highlighted. First, BU related treatment decisions are seen to be to be largely pragmatic when one considers the well-being of the household as the primary unit of analysis and not individuals afflicted with the disease. This is not to say that ill individuals are neglected by their households, but that basic HHPH needs eclipse individual medical needs. Second, practical logic supersedes biomedical reasoning when hospital-based treatment requires a mother to be displaced from her household. Removing a mother places the household at risk unless adequate substitute labor can be secured. Even then, the opportunity cost of a woman not being able to engage in resource generating activities constitutes a threat to her household. It also diminishes her ability to support others with whom she shares bonds of reciprocal exchange and mutual assistance.

A third lesson pertains to social capital as essential for survival among those living on the margin. Women in Benin constantly balance the need to invest in activities that provide material goods for their own household’s well-being with the need to convert time and material resources into social capital through helping kin in times of need. In the case of BU, offering support to kin is a means of accruing bonding social capital. However, the support a woman can offer is contingent on her carrying capacity (how many people she supports) and a husband’s ability and willingness to make contributions to the household. In Benin, male contributions to the household are unpredictable if a husband is working outside his community for months at a time.

A fourth lesson relates to health care decision-making and women’s agency. In the cases presented, it was largely men and their kin who made initial decisions about where BU treatment would be received. However, it was generally up to women to mobilize support from within their own kin networks to enable therapy management. Women’s agency and ability to influence decision making over time came from the resources at their command and the resources they could mobilize.

A fifth, related lesson is that social support is conditional. There are limits to the amount and duration of support kin can provide. In cases of BU treated in hospital, resource sharing and the constitution of TMG membership shifted over time. The amount of time needed to heal BU related wounds is difficult to predict, and hospital staff are generally not very forthcoming when it comes to communicating to a patient about how their healing process is progressing [17]. As a result, hospital patients are not sure what to tell family members when they inquire. Advanced BU patients often have to remain in hospital far longer than family members and caretakers imagined.

The inability to predict how long BU treatment might take in hospital settings placed strains on social relations. Women often expected more support than they received based on their assistance to others in the past. This was clearly seen in cases of diminishing support and abandonment. Diminishing support caused women considerable distress not just about the present moment, but the likely future. When their social capital was expended, they had no safety net.

A sixth lesson concerns the many risks hospital-based BU treatment poses for women in what might be thought of as a hierarchy of risks that encompasses physical, economic, social, and supernatural risks [45,55]. Three primary and multiple secondary risks face women. The three primary risks identified in this study are: risks to household economic survival associated with their displacement, risks to non-afflicted children left in the care of others, and the social risk to the reputation of a mother or daughter when they are compelled to reside in the liminal space of a hospital.

A seventh lesson is that whenever possible, decentralized outpatient treatment of BU is favored over hospital care. Decentralized treatment avoids the indirect and opportunity costs of “free” hospital care, allows an afflicted child and one’s other children to stay in school, and enables agricultural labor and income-generating activities to continue to the extent possible. It also reduces the risk of pernicious rumor, especially when community leaders visibly support community-near outpatient care. Visiting local health stations further avoids the serious problem of social isolation commonly felt by patients and caretakers in hospital, minimizes marital stress, and reduces fear of abandonment [29].

An eighth and related lessons is that decisions to go for decentralized BU care are not solely based on the accessibility of points of care. It is well documented that access to health facilities is a major factor affecting health care seeking [56]. It is certainly the case, that visits to traditional healers for chronic wounds are in part due to their close proximity. However, our research suggests that even when a health station offering decentralized treatment was equidistant to a BU reference hospital, people still preferred going to the health station for all but the most serious cases of BU. Data from a pilot project in Ouinhi commune confirm this observation.

In a recent publication[31] we reported on a pilot study that introduced decentralized treatment in Ouinhi commune. Notably, when decentralized care was introduced by a nurse with special training in chronic wound management, many households that had refused centralized BU treatment agreed to outpatient treatment at a local health station once they became aware of the service. In the case of Djalil this was due to convenience and not wanting to take his son out of school. In the cases of women we interviewed who had made a similar choice; it also had to do with diminishing social risk following an outreach program. Following the introduction of mass BU outreach education programs attended by chiefs, healers, and health staff, women traveling daily to get decentralized treatment were not subject to rumor. While the hospital was deemed a liminal space where behavior was suspect, the health station was seen as a valued community-near institution.

A ninth lesson entails decisions made about which family member should serve as a patient caretaker when hospitalization is required. This is one of the first articles to draw attention to young children serving as patient caretakers in West Africa for both their siblings and their mothers [57,58]. Children are chosen to be caretakers for two reasons. First, they enable a mother to remain at home to take care of other children and household affairs. Second, prepubescent girls are chosen because they are less likely to be the object of rumors about sexual liaisons in the hospital.

This brings us to the tenth and final lesson, the need for in-depth qualitative research, beyond surveys, as a means to more fully understand predisposing, enabling, and service related factors influencing health care and patient caretaker decision-making. Two examples may be highlighted: social risk and mothers’ concern about quality of childcare. As already noted, women recognized the danger of being treated in hospital posed by rumor. Adult women under treatment for BU, or functioning as patient caretakers, almost universally brought young children with them. They did so both to care for these children and to buffer them against pernicious rumor. Notably, during semi-structured interviews, childcare issues were commonly mentioned as a reason for sending young daughters to serve as caretakers, but concerns about rumors were not mentioned. These concerns only emerged during illness narratives, which may be the reason that the importance of social risk, moral identity, and rumor have been little discussed in studies that have relied on surveys and structured interviews that have not specifically queried this subject.

A second example involves substitute childcare as a significant factor in women’s decision making about hospital treatment. Mothers carefully consider the quality of care a daughter, grandmother, sister, co-wife, or foster parent might provide their children. Notably, their assessment is not just based on whether the person can provide food and shelter. Emotional support is also quite important. It was only during the collection of illness narratives that quality of childcare emerged as a primary health concern. In cases where good quality of care was not deemed available, mothers either opted not to seek hospital care for a child afflicted with BU at the expense of the well-being of their siblings, or took her children with her to hospital. Taking multiple children to a hospital has led to all kinds of logistical issues for hospital administrators.

### Limitations

A qualitative study utilizing a purposeful sample is designed to identify the range of factors effecting phenomena: in this case decision making about BU treatment and the experience of patients and caretakers in hospital. The study was not designed to measure which factors are most responsible for treatment delay, drop out, or no show after BU identification. This will require a quantitative study, which measures the order of magnitude of factors identified in this study.

### Conclusion

In this paper, we have used an ethnographic study of BU to make a case for focusing on the household as a unit of analysis when studying NTD related health care decision-making and its sequela in LMICs. Toward this end, we have found three conceptual lenses to be particularly useful: HHPH, gender relations, and TMG mobilization. Use of these lenses broadens our understanding of factors influencing treatment choice and patient caretaker selection when hospitalization is required. They also provide us with a nuanced account of the ripple effects of longstanding illnesses like BU beyond the household, and an appreciation that social support from kin is contingent and conditional.

In the areas of Benin studied, like many other regions of West Africa, men are the primary decision makers for healthcare decisions outside the home. A woman’s agency and ability to influence the decision-making process is largely based on whatever social support and substitute labor she can mobilize from her own network of kin relations. The brief BU case vignettes presented in this paper speak to the importance of bonding social capital for women in times of illness, describe ways in which this capital is accrued through reciprocal assistance, and draw attention to strains on social relationships when the duration of support needed is longer than expected and/or indeterminate. The three lenses further help us identify groups at risk for treatment delay, non-adherence, and patient abandonment at hospital due to structural vulnerability.

In conclusion, we argue that public health programs for diseases requiring long-term treatment, like BU, need to take into consideration household survival and gender relations and not just the medical needs of the afflicted. We concur with Grietens et al.[29] who argue that public health programs need to recognize the folly of designing programs that save the patient at the cost of compromising the integrity of the household and the health and well-being of other household members, especially children.

## Supporting information

S1 Fig Child caretaker: This five year old girl is the primary caretaker for both her mother, a hospitalized Buruli Ulcer patient, and her eighteen month old sister. (JPG)
