# Supplementary figures and images for: The gendered impact of Buruli ulcer on the household production of health and social support networks: Why decentralization favors women
Source: PLoS Negl Trop Dis. 2019 Apr 15;13(4):e0007317. doi: 10.1371/journal.pntd.0007317 (PMC6483275; doi:10.1371/journal.pntd.0007317)

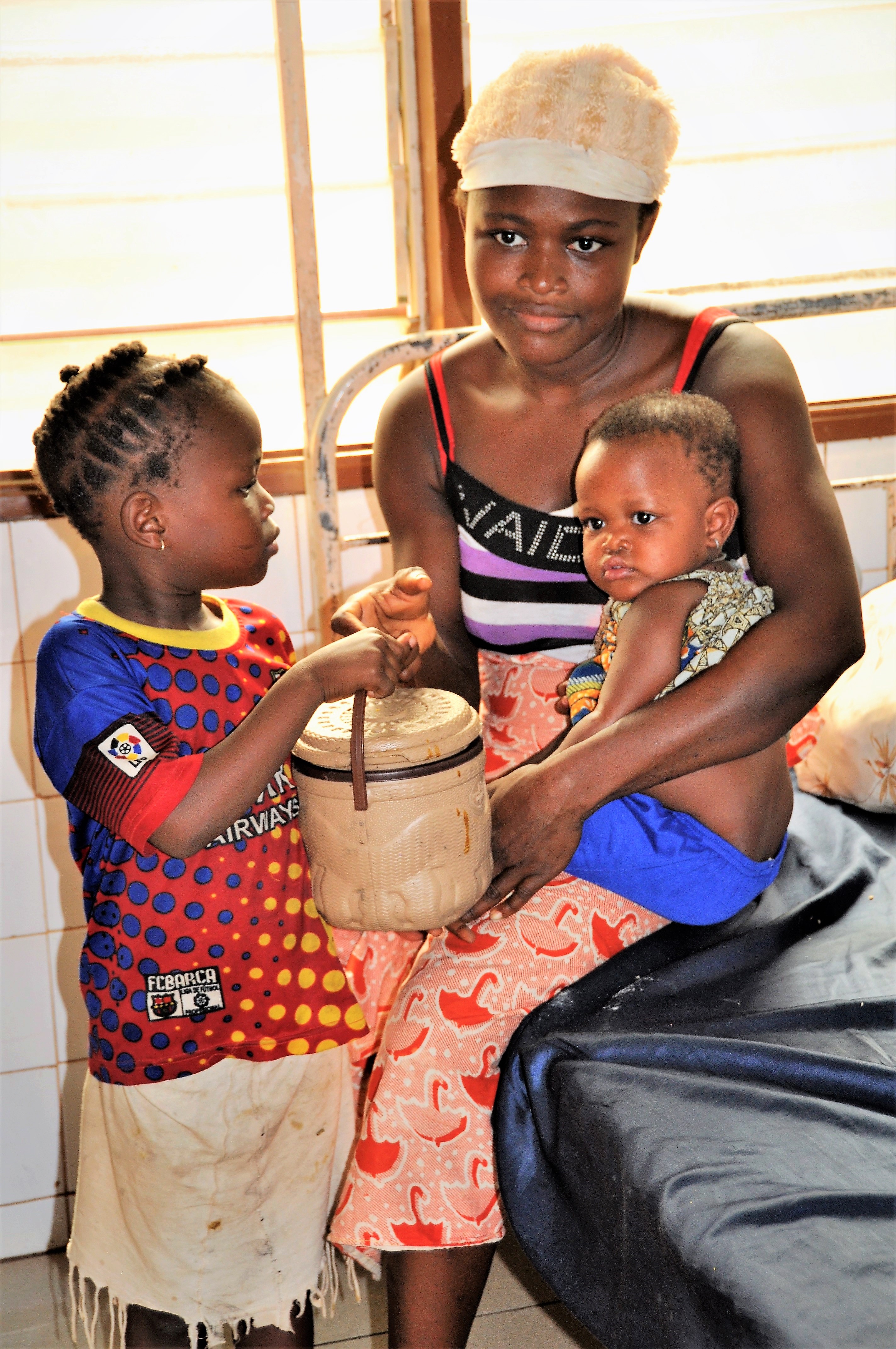

Supplement: S1 Fig — (JPG) [file pntd.0007317.s001.jpg]
